# Physiological Role and Use of Thyroid Hormone Metabolites - Potential Utility in COVID-19 Patients

**DOI:** 10.3389/fendo.2021.587518

**Published:** 2021-04-26

**Authors:** Eleonore Fröhlich, Richard Wahl

**Affiliations:** ^1^ Department for Diagnostic Laboratory Medicine, Institute for Clinical Chemistry and Pathobiochemistry, University Hospital Tuebingen, Tuebingen, Germany; ^2^ Center for Medical Research, Medical University Graz, Graz, Austria

**Keywords:** triiodothyronine, non-thyroidal illness syndrome, COVID-19, 3,5-diiodothyronine, tetraiodoacetic acid, triiodoacetic acid, thyromimetics

## Abstract

Thyroxine and triiodothyronine (T3) are classical thyroid hormones and with relatively well-understood actions. In contrast, the physiological role of thyroid hormone metabolites, also circulating in the blood, is less well characterized. These molecules, namely, reverse triiodothyronine, 3,5-diiodothyronine, 3-iodothyronamine, tetraiodoacetic acid and triiodoacetic acid, mediate both agonistic (thyromimetic) and antagonistic actions additional to the effects of the classical thyroid hormones. Here, we provide an overview of the main factors influencing thyroid hormone action, and then go on to describe the main effects of the metabolites and their potential use in medicine. One section addresses thyroid hormone levels in corona virus disease 19 (COVID-19). It appears that i) the more potently-acting molecules T3 and triiodoacetic acid have shorter half-lives than the less potent antagonists 3-iodothyronamine and tetraiodoacetic acid; ii) reverse T3 and 3,5-diiodothyronine may serve as indicators for metabolic dysregulation and disease, and iii) Nanotetrac may be a promising candidate for treating cancer, and resmetirom and VK2809 for steatohepatitis. Further, the use of L-T3 in the treatment of severely ill COVID-19 patients is critically discussed.

## Introduction

**Figure 1:**
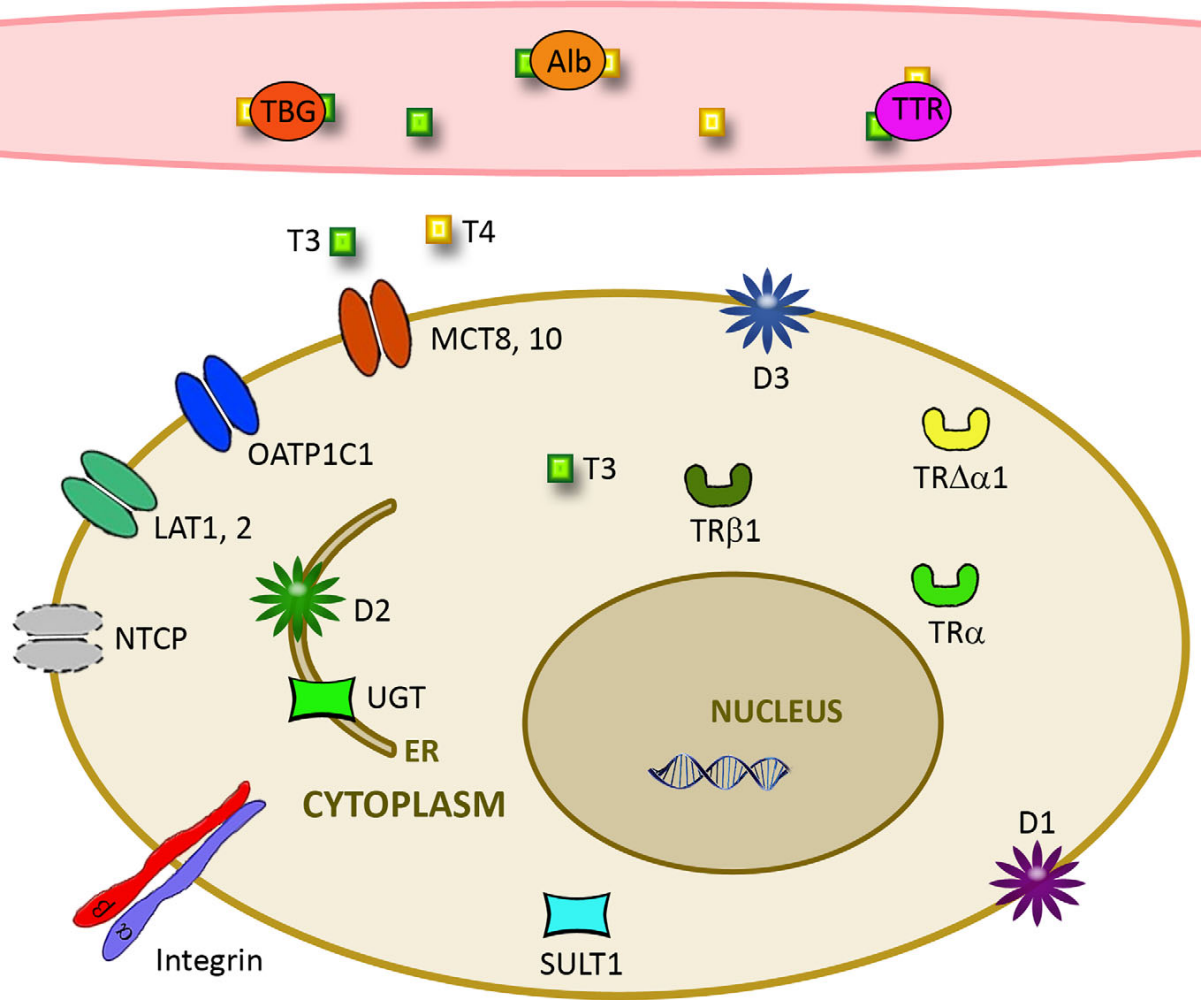
Important parameters for cellular effects of thyroid hormones. Free TH levels are influenced by the amount of distribution proteins such as albumin (Alb), thyroxine-binding globulin (TBG), and transthyretin (TTR). Cellular uptake is determined by expression of transporters like L-amino acid transporter (LAT), monocarboxylate transporter (MCT), Na+/taurocholate cotransporting polypeptide (NTCP), and organic anion transporting polypeptide (OATP). Deiodinases D1-3 together with Sulfotransferases (SULT1) and UDP-glucuronosyltransferases (UGT) determine action on the cellular level. Cellular effects are further influenced by the level of αvβ3 integrin and truncated and nuclear receptors TRα and TRβ.

Thyroid hormones (TH) are endocrine hormones that influence nearly all cells of the human body. Deficiency and excess, hypothyroidism and hyperthyroidism, demonstrate the action of TH on fetal development, lipid and carbohydrate metabolism, growth, cardiovascular, central nervous, and reproductive systems. TH action is determined by the level of circulating hormones and their metabolites, serum binding (distribution) proteins, cellular transporters, type and amount of deiodinases (D), and expression of receptors (**Figure 1**). Treatment with endocrine hormones is usually restricted to supplementation in case of insufficient endogenous production (e.g. in autoimmune diseases) or decreased target sensitivity (e.g. insulin resistance). TH may however also be candidates to treat specific conditions, like obesity, diabetes mellitus type 2, and metabolic syndrome. TH analogues with antagonistic effects may be helpful to support recovery from illness. Biological issues like lack of selectivity of TH action and finding suitable pharmaceutical formulations pose problems for treatment. Numerous recent studies focused on the role of pre-existing morbidities for risk of infection with Severe Acute Respiratory Syndrome-Coronavirus-2 (SARS-CoV-2) and severity of corona virus disease 2019 (COVID-19). Although thyroid dysfunction appears to play no important role as risk factor for contracting COVID-19 disease, alterations in TH levels were observed in hospitalized patients.

This review starts with an overview of production, transport, uptake, cellular metabolism and action of the classical representatives T3 and T4 and proceeds with a description of the biologically active TH metabolites. Although all of these aspects are covered by excellent reviews, this more practically oriented description of the different aspects influencing TH action has been included to illustrate the variety of factors that affect TH action and point out differences between the metabolites. One section is devoted to the role of TH in COVID-19. Finally, potential medical applications of TH metabolites and problems in the pharmacological formulation of these agents are addressed.

## Blood Transport, Metabolisation, Cellular Uptake, and Cellular Effects of TH

Structurally, TH belong to the amine hormones, which are derivates of amino acids such as tyrosine, tryptophan, and histidine. They include catecholamines (epinephrine and norepinephrine), melatonin, and histamine. The molecules generally are highly water-soluble, bind to membrane receptors and act *via* G-proteins, adenylcyclase, c-AMP, and protein kinases. TH differ from the general rule in the way that they are lipophilic and cause effects *via* both nuclear and membrane receptors (1).

Proteins influencing the cellular action of classical TH are shown in **Figure 1** and described in the following sections.

### Production and Blood Levels

Production of TH by the thyroid is 85 µg/day for T4 and 6.5 µg/day for T3. The majority of the estimated total amount of 30 µg T3/day is produced outside the thyroid parenchyma *via* T4 deiodination, mainly by deiodinases. TH levels show circadian rhythmicity with peak values of T4 from 8-12 am and lowest levels from 10 pm-3 am. T3 levels are highest from 7 am-1 pm and lowest from 11 pm-3 am (2). Levels are linked to those of thyroid-stimulating hormone (TSH), which precede them by around 6 h (peak at 2-4 am and nadir at 4-8 pm) (3). These data suggest a link of TH levels to metabolism and arousal. Overall, fluctuations of T4 and T3 levels in serum are not prominent, while tissue concentrations of T3 can vary dramatically (4). Regulation of TH levels through the hypophysis - pituitary gland – thyroid (HPT) axis is the main mechanism of TH secretion. The mechanism is described in several reviews dedicated to this topic [e.g. (5, 6)] and will not be addressed here. Age and sex influence TH levels and free TH but not TSH concentrations decrease in men with age, while in women the free TH levels remain constant but TSH level increase in an age-dependent manner (7). Decreased monocarboxylate transporter (MCT)8 expression and decreased deiodinase (D)1 activity in aged livers increase TH receptor (TR)β protein and shift T3 activity from liver to kidney (8). Reports of a correlation of low fT4 and longevity lead to the hypothesis that a slower metabolism with reduced production of oxygen radicals results in reduced cell damage and longer life. Prominent changes were seen during pregnancy when T4 levels increased sharply between week 6-9 and more slowly thereafter, resulting in stable values between week 20-27 of gestation. Increases in TH during pregnancy are accompanied by increase of thyroxine binding globulin (TBG) levels in blood due to longer half-life of the protein. fT4 and fT3 levels, the important parameters for TH action in the tissue, remain in the normal range (9). TH are important for fetal development and more detailed information on pregnancy-related changes in TH levels is available elsewhere [e.g. (10, 11)].

### Transport in Blood by TH Distributor Proteins

TH are transported in blood bound to transport proteins. For T4 the binding is 75% to TBG, 20% to transthyretin (TTR, prealbumin), and 5% to serum albumin. Apolipoprotein B and apolipoprotein A1-containing lipoproteins, contribute with 3% of T4 and 6% of T3 to TH transport. In the rare case of familial dysalbuminemic hyperthyroxinemia and hypertriiodothyroninemia higher binding of T4 and T3 may occur (12). The affected individuals have higher T4 and T3 levels but do not have any symptoms because fT4 and fT4 are in the normal range. Only a small fraction of 0.03% of T4 and 0.3% of T3 is circulating in free form in the blood (13). The main source of all distributor proteins is the liver but choroid plexus and retinal pigment epithelium are additional sources for TTR. Albumin and TTR are produced in syncytiotrophoblast cells of the placenta during pregnancy (14). Affinity of T4 to distributor proteins is higher than that of T3 and binding affinity for T4 and T3 increases in the order albumin<TTR<TBG (15). In contrast to what was initially hypothesized, limited solubility of T4 is not the reason for the need for distributor protein binding because solubility of T4 is higher than fT4 levels. The difference is impressive as the maximum solubility of T4 at pH 7.4 is 2.3 μM, while concentration of free T4 in human blood is only 24 pM. According to the current hypothesis, binding to distributor proteins serves as a buffer for T4 and provides a more even tissue distribution of the TH. Of the three most important proteins albumin, TTR, and TBG, TTR is responsible for most of the delivery because TBG binds T4 too tightly to allow release. Binding affinity of TH to albumin, on the other hand, is too weak and the protein is not efficient for distribution. The importance of TTR is corroborated by the fact that its absence of TTR is the only distributor protein pathology not compatible with human life, while absence of albumin and TBG deficiency does not create major symptoms. TTR is not only a binding protein for TH, it has many other biological functions (16). Only in mammals, TTR has higher affinity for T4 than for T3. Although intracellular T3 cannot be determined by blood analysis, measurements of T4, T3, and TSH are the commonly used parameters to assess thyroid function (https://www.thyroid.org/thyroid-function-tests/).

### Membrane Transporters

Entry into cells can be passive based on the lipophilicity of the molecules but also by transporters. The amphipathic nature due to the lipophilic aromatic rings and hydrophilic amino acid side chains may be the reason why thyroid hormones are suboptimal candidates for passive diffusion. The most important transporters of TH are MCT8, MCT10 and organic anion transporting polypeptide (OATP) 1C1. Further, L-amino acid transporters (LAT) 1 and 2, multidrug resistance-associated proteins (MDP), Na+/taurocholate co-transporting peptide (NTCP) 1 and fatty acid translocase (FAT) are involved (17). MCT10 transports T3 better than MCT8, while for T4 transport MCT8 is better than MCT10 (18). MCTs are the only exclusive TH transporters and are abundantly expressed in liver, intestine, kidney, and placenta. Both can transport TH into and out of cells but the relevance of export is unclear. OATP1C1 preferentially transports T4 and rT3 over T3, and is expressed mainly in the brain (4). LAT1 transports several TH metabolites in the order 3,3’-T2>T3~rT3>T4. The presence of multiple membrane transporters appears particularly relevant in Allan-Herndon-Dudley Syndrome (AHDS), where decreased numbers of oligodendrocytes in the brain correlates with TH due to mutation of the MCT8 and contributes to the pathology (19). As OATP1C1 is not highly expressed in the pre- and perinatal human brain, MCT10, LAT1 and LAT2 are the most likely candidates for the basal TH supply in these patients.

Importers and exporters in the nuclear membrane regulate the transport of TR from cytoplasm to nucleus (20). Mutation of the transporter proteins of TRα (importins α1, β1, and 7) and of TRβ (importin α1/β1 heterodimer) may play a role in TH resistance. Exportins 4, 5, 7, and calreticulin/exportin 1 complex are mainly involved in export from the nucleus.

### Deiodinases

There are 3 enzymes acting as deiodinases with different specificities, cellular localization, and organ distribution. The important effect of TH regulation by deiodinases on cellular levels is best illustrated by the reciprocal actions of D2 and D3 regarding energy expenditure (21). c-AMP induction of D2 expression in brown adipose tissue upon cold exposure increases energy expenditure, while hypoxia-inducible factor (HIF)-1α−dependent induction of D3 in myocardium and brain ischemia decreases expenditure. If the energy sparing is exaggerated, activation of D3 can lead to non-thyroidal illness syndrome (NTIS), also termed euthyroid sick syndrome. NTIS is characterized by elevated rT3 and low total T3 and fT3. TSH and fT4 can be normal but mortality increases steeply when both parameters, as in critically ill patients, decrease [(22), see also in the section *Reverse rT3*]. The condition is viewed as a protection mechanism to reduce energy expenditure that does not require treatment, while other groups recommend treatment with thyrotropin-releasing hormone (TRH), TSH, or T3 and T4.

All three deiodinases are selenoproteins but have different specificities. D1 is localized at the plasma membrane and exists in two forms, and can increase and decrease T3 levels. D2 localized in the endoplasmic reticulum provides T3 for receptor activation in the tissue, and D3 at the plasma membrane metabolizes T4 to rT3 and degrades T3 to T2 (23). Due to the fast binding to ubiquitin with subsequent proteasomal degradation, D2 has a much shorter half-life (30 min) than D1 and D3 (12 h). D1 catalyses 5’-deiodination (D1_1) as outer ring deiodinase (ORD) and converts T4 to T3 and rT3 to 3’,5’-T2 (24). Substrate affinity decreases in the order rT3>>T4≈T3. The D1 form for 5-deiodination (D1_2), also known as inner ring deiodinase (IRD), converts T4 to rT3 and T3 to 3,3’-T2. D1_1 has a greater affinity to T3 than to T4 and inactivation of T3 prevails over conversion of T4 to rT3. The high affinity to rT3 and sulfated thyrothyronines led to the assumption that the main function of D1 is to recover iodide from inactive compounds. In addition to Se deficiency, illness, specific drugs, cadmium, mercury or lead intoxication, and stress shift the balance to rT3 generation. D1 enzymes are abundant in liver, kidney and skeletal muscle (25). D2 catalyses the same reaction as D1_1 and is located in brain, pituitary gland, and brown adipose tissue. The affinity to T4 is greater than for rT3. A variety of signals, bile acids, flavonols, chemical chaperones, insulin, and peroxisome proliferator activated receptor (PPAR)-γ, induce D2 expression, while endoplasmic reticulum stress and liver X receptor/retinoid X receptor (LXR/RXR) activation dampen the D2 pathway. D3, with substrate affinity T4>T3 and function for T3 degradation, is similar in activity to D1_2 and found in the central nervous system. From its localization at the plasma membrane D3 may migrate to the nuclear membrane in ischemia, where it inactivates T4 and T3 (26).

Effects of polymorphisms of deiodinases on thyroid metabolism have been studied, but with controversial results. Carriers of the D_1b_-G/T(rs12095080) allele in D1 had higher T3 and T3/rT3 ratio, while carriers of D_1a_-G/T(rs11206244) allele had increases in fT4 and rT3 and decreases in T3 and T3/rT3 ratio. The affected individuals, however, presented no symptoms of thyroid dysfunction (4). An indication of interference with thyroid function has been reported in carriers of Thr92Ala polymorphism in D2, which need higher L-T4 concentrations to achieve euthyroidism and show delayed T3 secretion in response to TSH.

Regulation of TH on the cellular level includes conjugation. Sulfotransferases are located in the cytoplasm particularly of liver, kidney, and brain. Several members of the sulfotransferase (SULT) 1 family, namely SULT1A1, SULT1A2, SULT1A3, SULT1B1, and SULT1C2 catalyze conjugation with velocity 3,3’-T2> T3~ rT3> T4 (24). Glucuronidation of TH is performed by members of the Uridine 5’-diphospho-glucuronosyltransferase (UDP-glucuronosyltransferase (UGT)1A family, located in the endoplasmic reticulum. Tetrac and Triac are more rapidly glucuronidated in the liver than T4 and T3.

### Mechanisms of Cellular Action by TH

**Figure 2:**
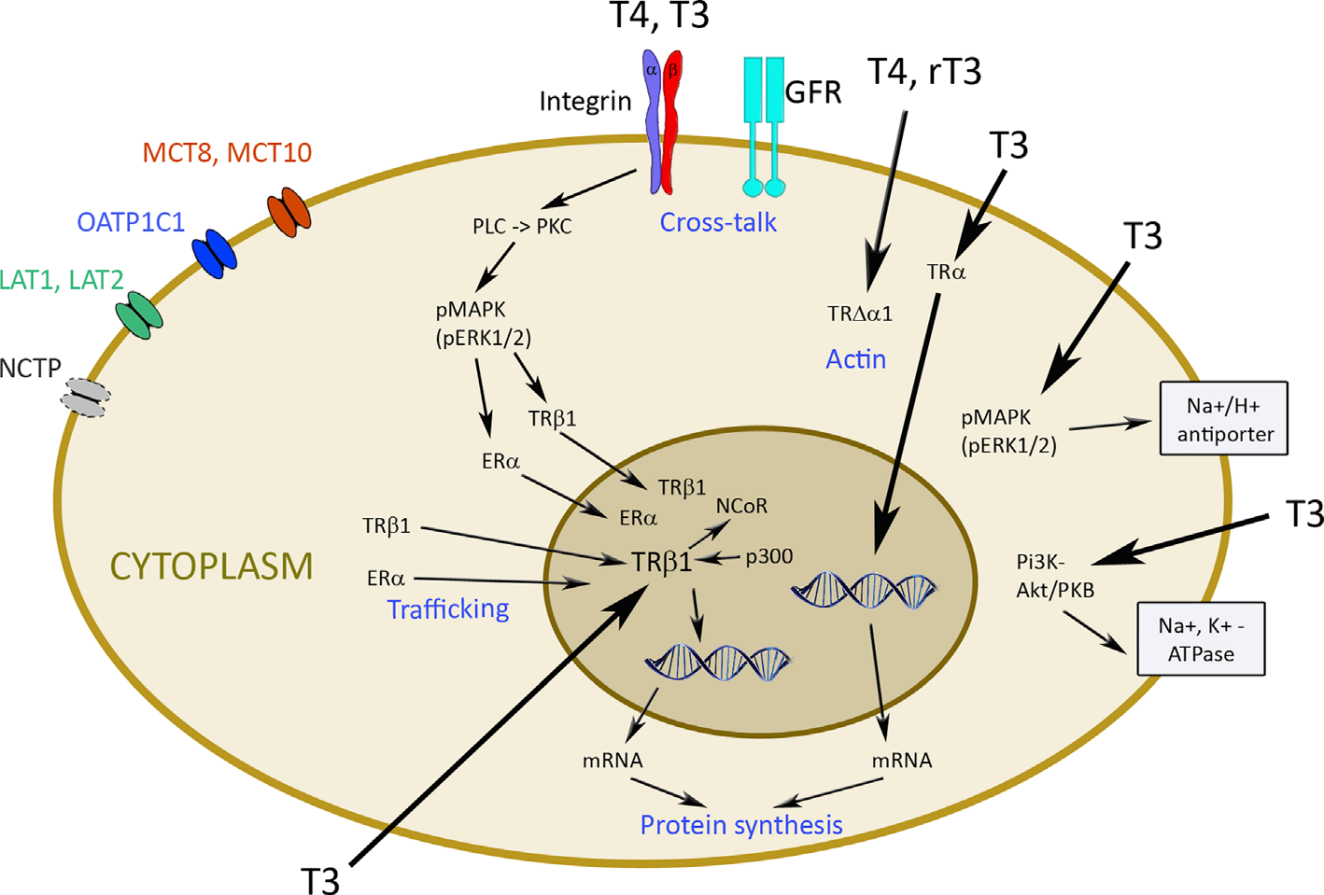
Transporters and mechanisms of genomic and non-genomic actions of thyroid hormones. TH enters cells by diffusion or *via* transporters like monocarboxylate transporter MCT8 and MCT10, organic anion transporting polypeptide (OATP)1C1, L-amino acid transporter (LAT) 1 and 2, and Na+/taurocholate cotransporting polypeptide (NCTP). Hormones inside the cell can bind to thyroid hormone transporter (TR) α and β1 and induce genomic changes *via* transcription. Nuclear receptor co-repressor protein (NCoR) and Adenovirus early region 1A binding protein p300 (p300) are involved in this process. Non-genomic regulation occurs by binding to integrin αvβ3 with activation of phospholipase C (PLC), protein kinase C (PKC), phosphorylated mitogen-activated protein kinase (pMAPK), and extracellular signal-regulated kinase (ERK) to induce trafficking of the phosphorylated TRβ1 and estrogen receptor (ER) into the nucleus. Cross-talk of αvβ3 integrin with growth factor receptors (GFR) is particularly important for cancer cells. T4 and rT3 can also act *via* TRΔα1 on actin, and T3 through pMAPK/ERK1/2 on Na^+^/H^+^ antiporter and *via* phosphatidyl-inositol 3 kinase (PI3K) and Akt/protein kinase B (PKB) on Na^+^/K^+^-ATPase.

In some mammalian species, a cytosolic T3-binding µ-crystallin was identified. According to rodent studies, ketimine reductase µ-crystallin, which is the human homologue, may mediate the intranuclear transport of T3, but allosteric regulation of enzymatic activity of the reductase by T3 also appears possible (27). Non-genomic regulation was discovered later than genomic regulation. Several reviews are available for detailed information on the complex and variable regulation of TH action [e.g. (28–30)]. Diversity exists on the receptor level because due to multiple splicing, multiple nuclear TRs, designated α1-α3 and β1-β3, and the truncated forms Δα1, Δβ2, and Δβ3 can be generated (31). The α2 and α3 isoforms and all truncated TRs are non-T3 binding proteins and function as antagonists of TH signaling. As a general rule, TRβ1 is mainly expressed in tissues linked to regulation of metabolism, while TRα1 is the major isoform in the heart. TRβ1 is highly expressed in liver, TRβ2 is particularly relevant for brain, retina, and inner ear, and TRβ3 is expressed in rodent tissues but not in humans (32). Action of nuclear receptors on transcription can be briefly described as follows (**Figure 2**). TRs form homo- and heterodimers with retinoid X receptors (RXR). Unliganded TRs bind to TH response element sequences in T3 target genes and mediate transcriptional repression. Co-repressor proteins (nuclear receptor co-repressor protein (NCoR)/homolog silencing mediator of retinoid) and TR are recruited to the RXR-TR heterodimer in the absence of T3 and inhibit target gene expression. The proteins contain three repressor domains and two receptor interacting domains (28). The receptor domains form a large repressor complex by interaction with different types of histone deacetylases (HDAC 1, 3) and transducin (beta)-like 1 (TBL-1). T3 binding causes a change in conformation with displacement of co-repressors and dissociation of the complex. Recruitment of co-activators results in interaction with RXR-TR heterodimer and transcription. Steroid receptor coactivator-1 (SRC-1) interacts with cyclic AMP (cAMP) response element binding protein (CREB) response element-binding protein (CBP) and Adenovirus early region 1A binding protein p300 (p300) through its activation domain 1 (AD1), with histone acetyltransferases through its AD2 and with ATP-dependent chromatin remodeling complex through its AD3 (33). The formation of such a coactivator complex results in chromatin remodeling and bridges the hormone-activated receptors with the general transcription machinery for transcriptional activation of their specific target genes. More information on the mechanism is available elsewhere [e.g. (28, 34)].

Non-genomic action of TH is initiated at the plasma membrane, in the cytoplasm (TRα or TRβ) or in intracellular organelles (e.g. mitochondria) (30) (**Figure 2**). Integrin αvβ3 represents one of the 24 integrin family members with an Arg-Gly-Asp (RGD) domain, which mediate interaction with extracellular matrix proteins, such as osteopontin, fibronectin, and vitronectin. The binding site of TH is distinct from this site and consists of the two domains S1 and S2. Binding to S1 is specific to T3 and S2 accepts T4 and, with lower affinity, T3 (28).

There is cross-talk between αvβ3 integrin and growth factor receptors [e.g. vascular endothelial growth factor (VEGF), endothelial growth factor (EGF), transforming growth factor β (TGFβ), insulin growth factor (IGF) 1) and basic fibroblast growth factor (bFGF)], which can be modulated by activation of extracellular signal-regulated kinase (ERK)1/2 (35, 36). Actions of αvβ3 integrin are mediated by two binding domains (28). Binding of TH to the S2 domain activates phospholipase C (PLC) and protein kinase Cα (PKCα), which leads to phosphorylation of TRβ, estrogen receptor (ER), signal transducer and activator of transcription 1 (STAT1), and p35. The phosphorylated TRβ1 translocates into the nucleus. Upon binding of T3 to the S1 domain, phosphatidyl-inositol 3 kinase (PI3K)/Akt/protein kinase B (PKB) pathway *via* Src kinase is regulated and Src kinase activation induces TRα1 translocation from the cytoplasm to the nucleus. The non-genomic regulation ensures independent function of the cytoplasmic and nuclear receptors (37, 38). TRΔα1 is involved in the maintenance of the actin cytoskeleton by T4 and rT3, and the p30 TRα1 peptide at the plasma membrane regulates T3-induced proliferation of non-malignant cells *via* ERK1/2 and Akt pathways. Binding of T3 in the cytoplasm to TRβ1 activates the PI3K/Akt/mTOR pathway, which then activates Na^+^/K^+^-ATPase. The T3-TRβ1 complex stimulates the Na^+^/H^+^ exchanger at the plasma membrane *via* MAPK/ERK 1/2 (28).

Due to the numerous interactions between non-genomic and classical TR signaling, Flamant et al. suggested a new classification system with four types of TH signaling based on involvement of TR and extent of interaction with DNA (39). Type I describes TR-dependent signaling with direct binding to DNA and can occur as monomer, homodimer or heterodimer (usually with retinoid X receptor, RXR) binding to response elements, TR binding to enhancer elements, and as a heterodimer with other partners, such as retinoic acid receptors. Type II is TR-dependent signaling with indirect DNA binding, where TRs interact with a number of chromatin-associated proteins. In type III, signaling is TR-dependent but no binding to DNA occurs. Interaction of cytoplasmic TR with kinases associated to the plasma membrane is the mechanism, and p30 protein translated from an internal codon of TRα1 is one example. In type IV signaling of TH no TR are involved and binding of TH to αvβ3 integrin regulates kinases, influences actin polymerization, and allosterically regulates metabolic enzymes.

## Relationship of SARS-CoV-2 and Thyroid

### Influence of COVID-19 on TH Levels

Based on a report of co-morbidities in COVID-19 patients, thyroid dysfunction is not a predisposing parameter for the disease. About 0.5% of the 7,162 patients reported had thyroid disease as a co-morbidity (40), which corresponds to a lower incidence than in the general adult population of the United States of America. Another study reported incidence similar to the general population (41). Both studies concurred that hypothyroidism is not a risk factor associated with worse outcome in COVID-19-positive patients (42) but thyroid disorders were linked to higher mortality of COVID-19 infected patients (43).

A variable percentage of COVID-19 patients was affected by thyroid dysregulation and different pattern of abnormalities were seen (**Table 1**). It has to be taken into account that not all studies used the same control groups and that the focus of the studies were different. While in one set of studies COVID-19 specific effects on the TH levels should be studied, in others the role of TH levels in the severity of the disease should be identified. In three studies, only a minority of COVID-19 patients presented with abnormal thyroid values (44, 45, 47). Inflammation markers (CRP, erythrocyte sedimentation, lactate dehydrogenase levels) correlated with reduced TSH and fT3 levels. Further, patients with low T3 had a higher risk for deterioration (44). Decreased TSH and T3 levels, isolated decreased TSH levels, decreased TSH and increased T4 levels, and decreased TSH and fT4 were reported. In the studies, where more than one third of COVID-19 patients had abnormal TH levels, decreased TSH and T3 was seen more often than decreased TSH in combination with increased T4 levels. In the group of the severely ill COVID-19 patients TSH and total T3 were reduced and the extent of T3 reduction correlated with the severity of the disease (48). It was further reported that decreases in T3 levels were more pronounced in the COVID-19 patients than in other critically ill patients. Lower TSH and total T3 levels in COVID-19 patients compared to patients hospitalized for non-COVID-19 pneumonia were also reported in two other studies (49, 51). Decreases in total T3 and TSH levels were correlated to increases in pro-inflammatory cytokines, such as interleukin (IL)-2, IL-6, IL-7, interferon (INF)-γ, and tumor necrosis factor (TNF)-α. T3 predicted all-cause mortality and IL-6 and C-reactive protein (CRP) were negatively correlated with fT3 levels. TSH and fT3 levels were also significantly decreased in deceased patients compared to recovered patients (52). Of note, differences in T4 levels were not statistically different in these groups. In the study published by Malik et al., by contrast, severely ill COVID-19 patients had increased T4, TSH, IL-6 and procalcitonin levels (50). The preprint evaluated a patient collective, where 85.7% of the controls (hospitalized non-COVID-19 patients) showed abnormal TH levels and where the incidence of hyperthyroidism with 4.5% was considerably higher than in Europe (0.7%) and the United States of America (0.5%). It may be assumed that the reported results from this single center study cannot be generalized.

**Table 1:** Overview of studies on prevalence and relevance of thyroid dysregulation in COVID-19.

Number of COVID-19 patients in the study	Patients with abnormal TH values (%)	Focus of the study	Type of alterations in TH levels; control groups	Reference
191	13.1	Specificity of TH level changes for COVID-19	TSH decreased (5.2%); fT3 decreased (5.2%); COVID-19 *vs* non-COVID-19 hospitalized patients	(44)
334	13.7	Specificity of TH level changes for COVID-19	TSH and fT4 decreased; COVID-19 *vs* non-COVID-19 hospitalized patients	(45)
52	21	Role of TH levels for the severity of COVID-19	TSH decreased and T4 increased (15%); critically ill COVID-19 *vs* critically ill non-COVID-19 patients	(46)
60	35	Role of TH levels for the severity of COVID-19	TSH and T3 decreased (18.3%); TSH decreased and T4 increased (9.1%); critically ill *vs* non-severe COVID-19	(47)
50	58	Specificity of TH level changes for COVID-19	TSH and total T3 decreased and T4 normal; COVID-19 *vs* non-COVID-19 pneumonia and healthy individuals	(48)
84	61.9	Specificity of TH level changes for COVID-19	TSH and total T3 decreased; COVID-19 *vs* non-COVID-19 pneumonia and healthy individuals	(49)
48	75	Specificity of TH level changes for COVID-19	TSH and total T4 increased, total T3 decreased; COVID-19 pneumonia *vs* non-COVID-19 pneumonia	(50)
100	na	Role of TH levels for the severity of COVID-19	TSH and fT3 decreased; critically ill *vs* to non-severe COVID-19	(51)
113	na	Role of TH levels for the severity of COVID-19	TSH and fT3 decreased: deceased COVID-19 *vs* survivors	(52)

Different mechanisms have been postulated to explain the observed alterations of TH levels in COVID-19 patients. Elevated IL-6 levels are typical for severe COVID-19 infections, while the concurrent increase in anti-inflammatory IL-10 is interpreted as response to overwhelming systemic inflammation (53). This interleukin has also be identified as key cytokine in NTIS and was identified as strong independent predictors of mortality in NTIS (54). FT3 levels in NTIS patients were negatively correlated to IL-10 levels but not associated with IL-6 levels. There is reason to assume that severely ill COVID-19 patients suffer from NTIS because IL-6 levels, which are generally increased in this condition, were identified as prognostic parameter for severity of COVID-19 infection (55).

The thyroid may, however, also be a target of the virus because expression of angiotensinogen converting enzyme 2 (ACE2) and the transmembrane protease serine 2 (TMPS2) is high (56). To compensate the lack of information about biological effects of SARS-CoV-2, data obtained from the closest related member of this virus family, SARS-CoV, are often used for estimating SARS-CoV-2 effects. Although no virus RNA was found in thyroid tissue, data from five autopsies of SARS-CoV patients showed apoptosis in follicular and interfollicular cells (57). This suggests that destructive thyroiditis may occur in COVID-19 patients. Subacute thyroiditis (SAT) can be caused by viral infections and manifests itself by thyrotoxicosis, followed by hypothyroidism and return to the euthyroid condition (58). The classical form is characterized by painful swelling of the thyroid and this was confirmed in few case reports of COVID-19 patients. Atypical thyroiditis, which does not manifest with pain and swelling, was seen in 15% of COVID-19 patients and could also explain an increase in T4 (46). To reveal a potential effect of the SARS-CoV-2 virus on the thyroid, thyrotoxicosis was evaluated in patients admitted to the high intensive care unit (HICU) in 2019 compared to 2020. In 2020, 15% of the patients had thyrotoxicity compared to 1% in 2019. By contrast, the rate of pre-existing thyroid disorders was lower in 2020 than in 2019, making thyroid disorders as risk factor for severe COVID-19 unlikely. Elevated T4 levels were seen in COVID-19 patients, but not the elevated T3 levels typically seen in viral SAT (46).

In addition to a direct attack on the thyrocytes, the virus may trigger destruction of the thyroid indirectly *via* cytokines (59). The authors observed thyrotoxicosis in 20% of COVID-19 patients admitted to the hospital. These patients presented with low TSH, increased T4 and normal fT3 levels in combination with high IL-6 levels. As has been shown in cancer patients treated with TNF-α and healthy individual after IV injection with IL-6, pro-inflammatory cytokines decrease T3 levels (60). Cellular studies indicated action on various steps of T3 synthesis, TNF-α further decreased synthesis of TSH. Changes of TH levels induced by the cytokines were similar to the pattern seen in NTIS.

Injury of TSH-producing cells of the pituitary gland as reason for the decreased TSH levels is also suspected (48). This theory is supported by the observation that number and intensity of the staining with anti-TSH antibody was decreased in pituitary glands of deceased COVID-19 patients. Decreased TSH levels may result from direct virus attack on TSH-producing cells in the pituitary gland or by action of cytokines (49).

Further explanation for the abnormal TH levels would be treatment with glucocorticoids (48). Glucocorticoids over a wide range of concentrations decrease TSH levels and inhibit the conversion of T4 to T3, while stimulating the conversion of T4 to rT3 (61). The induced pattern resembles NTIS. Since about 50% of COVID-19 patients are treated with glucocorticoids, this effect needs to be taken into account (60).

In summary, NTIS, elevated IL-6 and TNF-α levels, glucocorticoid treatment and hypophysitis may cause a similar pattern of decreased TSH levels, slightly reduced T4 and T3 levels. SAT and atypical thyroiditis, by contrast, would be characterized by transiently elevated T4 and T3 levels. After recovery normalization of TH levels without treatment has been reported for infections with the SARS-CoV and SARS-CoV-2, which occurs after both thyroiditis (SAT or atypical) and NTIS (50, 59, 62). The rate of hypothyroidism in 7% of patients after infection with SARS-CoV is in the same order as for SAT (62, 63). Indication of rT3 levels may help in differential diagnosis but this parameter is not routinely being determined.

It should also be mentioned that heparin, a prophylactic anticoagulation for hospitalized COVID-19 patients in hospital, can interfere with the measurement of free TH levels (60). The activity of lipoprotein lipase in blood samples is increased by heparin and non-esterized fatty acids are generated during sample storage. These acids displace T3 and T4 from their binding proteins and cause artificially high levels of the free hormones.

### Common Targets of SARS-CoV-2 and TH

#### Effects of SARS-CoV-2

Primary targets of SARS-CoV-2 are pneumocytes, immune cells, and vascular endothelial cells. Although most patients with COVID-19 manifest fever and respiratory tract symptoms, SARS-CoV-2 infection may also cause extra-respiratory symptoms, including cardiac, gastrointestinal, hepatic, renal, neurological, olfactory, gustatory, ocular, cutaneous and haematological system. Independent from the primary manifestation, death is generally associated with elevated levels of cytokines, interleukin IL-6, IL-1, and tumor necrosis factor (TNF-α), and COVID-19-associated coagulopathy (CAC) (64). Thrombotic complications were seen in one third of patients infected with SARS-CoV-2. Incidence of venous thromboembolism in COVID-19 patients was high, affecting 69% of patients admitted in the Intense Care Unit (ICU), and much higher than in other patients with acute respiratory distress syndrome (ARDS) (53). It is proposed that in the early phase of the disease, hypercoagulation predominates, and anticoagulation e.g. by heparin may be helpful. CAC presents specific differences to common coagulopathies like sepsis-induced coagulopathy and disseminated intravascular coagulation. Typical features of CAC are increased D-dimer levels, elevated inflammatory cytokines but minor changes in prothrombin time (PT), activated partial thromboplastin time (aPTT), and platelet count (65). D-dimer elevation was the most common finding and associated with severity and prognosis (66). Incidence of thrombocytopenia was seen only in 11% of SARS-CoV-2 infections and lower than for SARS-CoV infections (53). Blood levels of (vWF) and factors of the complement system are also increased. Increased release of van Willebrand factor (vWF) and FVIII, elevated expression of P-selectin by endothelial cells and damage of the glycocalyx of the endothelial cells has been reported in COVID-19 patients. These findings suggest endothelial dysfunction due to infection of the cells with the virus. Endothelial dysfunction affects regulation of vascular tonus, endothelial permeability, cell adhesion, and anticoagulation. The release of nitric oxide is particularly important for prevention of leukocyte and platelet adhesion, inflammation, migration of immune cells, smooth muscle cell proliferation and suppression of apoptosis.

#### Effects of TH

Circulating TH levels may improve or worsen the clinical situation based on their action on clotting, endothelial function and immune system. It has been reported that manifest hypothyroidism was linked to bleeding and hyperthyroidism to venous thromboembolism and that fT4 levels were associated with increased FVII, fibrinogen, and vWF levels (67). It is hypothesized that T4 acted by increased transcription of coagulation proteins by activation of TRβ. On the other hand correlated fT3 levels with FIX activity, but neither fT4, fT3 nor TSH with fibrinogen, antithrombin III (ATIII), tissue plasminogen activator (t-PA), plasminogen activator inhibitor 1 (PAI-1) or vWF (68). The association of hyperthyroidism with hypercoagulable states and moderate-to-severe hypothyroidism with hypocoagulable states appear to be caused by the respective fT4 levels and not associated with fT3 levels (69). Blood clots formed in hyperthyroid individuals showed a much denser fibrin network and were more resistant to fibrinolysis (70).

Increased rT3 levels found in NTIS, on the other hand, may promote hypocoagulable states in the patients because rT3 inhibits collagen-induced platelet aggregation. T4, on the other hand, promotes platelet aggregation and degranulation, and T3 appears to cause no effects in platelets (71). The lack of action of T3 on platelets can be explained by the fact that platelets are anucleate and do not have nuclear receptor proteins. Expression of αvβ3 integrin on platelets is lower than that of other integrins, and T4, in addition to αvβ3 integrin binding, may support pathologic platelet aggregation by regulation of CX3CL1 (Fractalkine) (71, 72). Fractalkine induces platelet adherence to collagen.

TH can further increase the interaction of platelets and endothelium through the αvβ3 integrin-adjacent receptor VEGF, increasing angiogenesis (70). Potential mechanisms include increased amount of CD31 (PECAM-1) or increased αvβ3 integrin/PECAM-1 binding. Oral addition of L-T4 to euthyroid individuals resulted in elevated expression of several coagulation proteins. TH status appears to play a role for the development of atherosclerosis, and subclinical hypothyroidism was found to be correlated with endothelial dysfunction, where increased TSH levels were hypothesized as mechanism of action (73). Application of L-T4 to women with subclinical hypothyroidism did not reduce intima-media thickness (74). L-T3, on the other hand, induced expression of endothelial nitric oxide synthase (eNOS) in human umbilical vein endothelial cells in the presence of IL-1β and acted vasoprotective in hypertensive rats by reducing ROS levels (75, 76). The proposed mechanism here is T3 binding to integrin αvβ3 followed by PI3K/Akt signaling and increased eNOS production (77).

The importance of T3 and T4 levels and the T3/T4 ratio in the euthyroid condition for the immune system has been studied by Hodkinson et al. (78). Higher T3 levels were associated with higher complement levels, increased phagocytic activity of monocytes, elevated natural killer (NK) cell counts, higher percentage expression of IL-6, and higher monocyte counts. Higher T4 levels were correlated to higher complement C3 and C4, C-reactive protein concentrations, neutrophil counts, and percentage expression of T memory cells (78). In combination with other studies, the following differences between T3 and T4 were identified. T3 in physiological concentrations increased NK cell activity and interferon (IFN)-γ responses on NK activity. Further, T3 stimulated maturation, functional activation, viability, and antigen cross presentation-allostimulatory capacity boosting antigen-specific cytotoxic T cell responses of dendritic cells (DCs) (79). T4 was unable to induce effects in DCs because only T3 was taken up by the cells. Similarly, NK cell activity was not increased, when T4 was applied to isolated lymphocytes. T4, 3,5-T2, and T3 increased respiratory burst activity, reactive oxygen species (ROS) levels, myeloperoxidase and NADPH oxidase activity of neutrophilic granulocytes, as well as ROS levels and phagocytosis of macrophages. Bacterial killing and pro-inflammatory response may be increased or decreased. In addition, T4 inhibited secretion of migration inhibitory factor (MIF) by macrophages, while no changes in IL-6 and TNF-α levels were detected. T4 increases expression of VEGF, ICAM-1, E-selectin, IL-6, and TNF-α in human umbilical vein endothelial cells (HUVEC) (80). It is suggested that inhibition of IL-6 signaling induced by T3 has potent regulatory functions during infection and inflammation, and decreased intracellular concentrations of T3 resulted in impaired polarization of macrophages into pro-inflammatory M1 type (81, 82). T3 stimulates immune reactions *via* action on lymphocytes and monocytes and supraphysiological levels of T3 decreased replication of vesicular stomatitis virus (83) and supplementation with T3 increased the number of resident (potentially beneficial) peritoneal macrophages in mice with endotoxemia (84). TRβ1 was identified as the major player mediating T3 effects on macrophages. Decreased T3 levels in NTIS most likely impair immune cell function, particularly action of the specific immune system leading to insufficient protection against pathogens, e.g. viruses. Predictive data on the reaction of the human immune system *in vivo* are difficult to obtain because evaluation *in vitro* cannot represent its complexity and species-specificity of the immune system limits the value of animal studies.

## Biological Effects of Metabolisation Products of TH

### Detection of Metabolisation Products

In addition to T4 and T3, various TH metabolites circulate in blood, some of which mediate biological effects. Exact levels are not known due to cross-reactivity of TH metabolites in conventional immunoassays, and it is also clear that metabolites found in plasma do not reflect tissue concentrations (85). Uncertainties of exact levels are not restricted to TH metabolites but exist also for T3, fT4 and fT3 levels. Liquid chromatography-tandem mass spectrometry (LC-MS/MS) is the reference method for exact TH quantification but shows a general trend for overestimation, particularly of free TH (86). Based on LC-MS/MS data, 48% percent of patients were classified as hypothyroid compared to 11% by immunoassay. In another study, in a cohort of 40 patients diagnosed with subclinical hypothyroidism (normal fT4 and increased TSH levels in conventional immunoassay), 65% had fT4 and fT3 levels below the reference level. A major reason for the better performance of LC-MS/MS in the determination of fT4 and fT3 is the removal of TH binding proteins during sample preparation.

**Figure 3:**
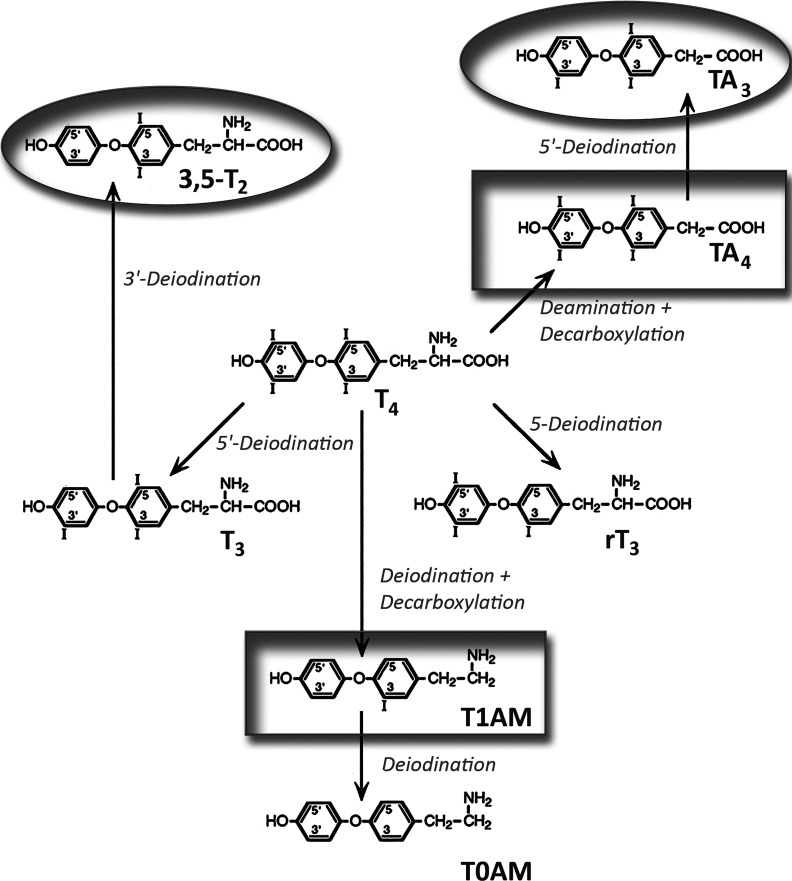
Generation of biologically active thyroid hormone metabolites from thyroxine. Circles mark agonists and boxes antagonistically acting metabolites. 3,5-T2, 3,5-diiodothyronine; T1AM, 3-iodothyronamine; rT_3_, reverse triiodothyronine; TA_4_, tetraiodoacetic acid; T0AM, thyronamine; TA_3_, triiodoacetic acid.

Important TH metabolites that circulate in blood include the iodinated derivates of the phenolic amino acid thyronine similar to T4 and T3, namely reverse T3 (rT3), 3,3’-diiodothyronine (3,3’-T2), and 3,5 – diiodothyronine (3,5-T2), but also tetraiodoacetic acid (Tetrac), triiodoacetic acid (Triac), 3-iodothyronamine (3-T1AM), thyronamine (T0AM), and the conjugated TH metabolites, TH sulfate, and glucuronide. T4 deiodination leads to rT3, and T4, T3, and rT3 deiodination to 3,3’-T2 and 3,5-T2, respectively. 3-T1AM and T0AM are formed by T4 and T3 deiodination and amino acid decarboxylation (**Figure 3**). Tetrac and Triac are produced by oxidative deamination and decarboxylation of T4 and T3.

### Reverse T3

The role of rT3 is receiving increasing interest because high levels are seen in NTIS, in contrast to low T3 and T4 levels. The TH metabolite is also increased in starvation, surgery, bone marrow transplantation, heart attack, coronary bypass grafting, and chronic dieting (87). Centenarians exhibit significantly lower TSH levels together with slightly higher rT3 levels than aged controls (7). rT3 binds to TBG with 40% higher affinity than T4. Also TTR and albumin plus several other serum proteins, in particular high density lipoproteins bind rT3 but contribution to overall transport is minimal.

Upon binding of rT3 to D1 or D2, biologically inactive 3,3-T2 is formed. Drugs like dexamethasone, propyl thiouracil, iopanic acid and sodium ipodate, amiodarone, and propranolol decrease T3 and increase rT3 levels (25) rT3 influences various cellular events, e.g. mitosis, cell migration and invasion, by conversion of soluble actin to fibrous actin. rT3 has a role in glial-mediated neuronal guidance during mammalian brain development but when rT3 is lacking, no prominent symptoms arise (85). Increases in rT3 are seen in physiological and pathological conditions. Serum T3 was significantly lower and serum rT3 significantly higher in diabetic patients prior to treatment as compared to normal subjects (88). Both T3 and rT3 normalized in 20 patients studied when adequate metabolic control was achieved as reflected by normalization of HbA1. Increased levels of rT3 also occurred in patients with myocardial infarction, hepatitis, or hepatic cirrhosis (89). On the other hand, insulin resistance was linked to an increased T3/rT3 ratio (90). All data suggest that the T3/rT3 ratio is decreased when metabolism is dysregulated, a notion supported by the finding that stress (induced by sleep deprivation, cold exposure, examination) and elevated cortisol levels (even if still in the normal range) are linked to raised rT3 levels (25). It appears that a rise in rT3 indicates short-term changes in metabolism rather than being a marker for chronic alterations because after 3 weeks of caloric restriction, T4 and rT3 levels returned to normal. The theory of rT3 being a marker for acute metabolic dysregulation is also compatible with the observed decrease in rT3 in hibernating bears. In this physiological long-term state, the bears showed a hypothyroid condition with decreased rT3 levels (91). Species-specificity, on the other hand, could also be the reason for the different reaction.

According to a generally accepted hypothesis, rT3 levels increase in specific conditions to conserve energy and to protect cells against insufficient T4, which could be converted to T3. rT3 is hypothesized to act as an energy-saving mechanism and as compensation for the decreased T3 levels (92). Upon dieting, T3 levels can decrease to 50% and after prolonged dieting not return to normal levels upon normal food intake. Conversely, in alternative day fasting normal T3 and rT3 levels are restored after a meal (93). During starvation more than the normal rate of 40% of T4 is converted to rT3 and less than the normal 60% to T3 with the effect of a changed rT3/T3 ratio. Increase of rT3 levels in dieting is mainly due to decreased elimination of rT3 during fasting, while decrease of rT3 after re-feeding is caused by decreased production. D1 was more sensitive to starvation than D2 and D3, which is consistent with the finding that changes in T3 affect mainly peripheral tissues, not the brain. This regulation serves the purpose of maintaining function of the central nervous system. Carbohydrate levels are important regulators in rT3 and T3 levels in low caloric intake between 360-1200 cal/day (94).

**Figure 4:**
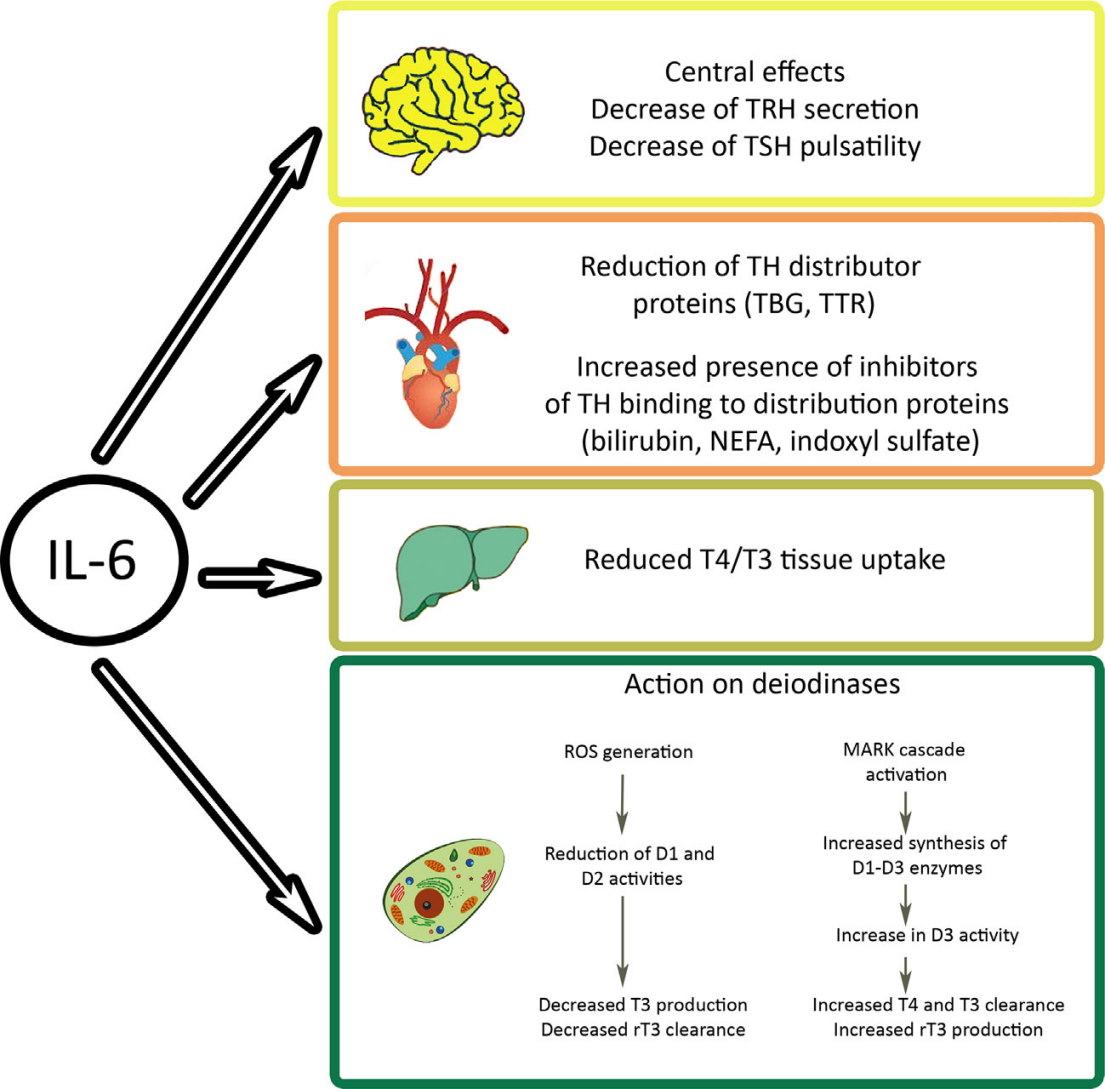
Effects of interleukin (IL)-6 on cellular thyroid hormone levels. The inflammatory cytokine acts on hypothalamus and pituitary gland of the brain, on levels of free hormones, on transporters, and on cellular deiodinases. Indoxyl sulfate is a metabolite of tryptophan that accumulates in kidney damage. NEFA, non-esterified fatty acids; TBG, thyroxine binding globulin; TRH, thyrotropin releasing hormone; TSH, thyroid stimulating hormone; TTR, transthyretin.

The most dramatic manifestation, where increased rT3 levels are seen, is NTIS. Also in this situation, the changes are interpreted as protection mechanism to reduce energy expenditure that does not require treatment, while other groups recommend treatment with thyrotropin-releasing hormone (TRH), TSH, or T3 and T4. In patients with NTIS, half-life of rT3 was short (3h) and levels mainly influenced by liver deiodinases (95). Davis et al. do not exclude the possibility that increased rT3 levels in NTIS contribute to hypercoagulation because rT3 acts similar to T4 on αvβ3 integrin (96). rT3 levels were negatively correlated to D1 in liver and positively to D3 in liver and muscle. The proposed mechanism of decreased action of D1 is downregulation by inflammatory cytokines TNF-α, interferon α, and IL-6. IL-6 plays a key role in the regulation of TH levels by acting on central and peripheral levels (**Figure 4**). IL-6 is increased in NTIS and levels inversely correlated (r=0.56) with T3 levels and positively correlated (r=0.78) with rT3 levels (97). Low T3 and T4 levels are detected extremely frequently in patients in intensive care, namely in 70% and 50%, respectively. Processes contributing to the findings in NTIS include IL-6 induced decrease of TBG and TTR, decreased T4/T3 tissue uptake, increased or unchanged transporter expression, decreased D1 and increased D3 expression in liver and increased D2 in muscle (98). Changes in acute and prolonged disease differ in the way that pulsatile TSH surge is present, TRH mRNA and T4 levels normal, T3 low and rT3 elevated in the acute, while TSH surge is absent, TRH mRNA and T4 levels low, T3 very low, and rT3 normal in chronic NTIS.

Circulating rT3 levels are also increased in cancer patients (96). rT3 increases proliferation of cancer cells *via* αvβ3 integrin signaling, which is highly expressed in such cells. It is supposed that cancer cells use this metabolite for proliferation instead of T3, which at physiological concentrations does not promote proliferation. This is in line with the finding that suppression of TH synthesis by methimazole and substitution by T3 in terminal patients induced stabilization or regression of the disease (71). Another, very rare, manifestation of increased rT3 levels is consumptive hypothyroidism seen in hemangioma (99).

### 3,5 Diiodothyronine (3,5-T2)

Of the several diiodothyronines, biological effects have been identified for 3,5-T2, while other diiodothyronines have higher serum levels (3,3’-T2: 1-8 ng/dl; 3’,5’-T2: 1.5-9 ng/dl) but no effect on metabolism (100). 3,5-T2 has blood levels of 0.2-0.75 ng/dl and low binding to TBG (101). Other studies reported up to 15 ng/dL, and blood levels have to be interpreted with caution because interference between diiodothyronines and T3 occurs (85). Inter-individual differences in 3,5-T2 levels have been reported, which are not linked to T3 levels. In NTIS, 3,5-T2 correlated with rT3 and may result from either increased production from T3 or decreased degradation. The 3,5-T2 levels increased by 30% in NTIS and may be seen as a balance to the increased rT3, that has opposite effects on metabolism (102). It is assumed that increased 3,5-T2 levels in otherwise healthy individuals may be an indication of an underlying disease. 3,5-T2 can bind to the T3 receptor and induce TSH suppression and alter expression of classical T3-regulated genes in liver and other tissues. 3,5-T2 increases mitochondrial oxygen consumption in rats rapidly (103). Increased 3,5-T2 levels in disease have been interpreted as compensation for low cellular T3 levels (104). Survival in chronically cold-exposed animals is improved due to activation of thermogenesis, stimulation of β-oxidation, and up-regulation of F(0)-F(1)-ATP synthase. In HepG2 hepatocytes, 3,5-T2 blocked proteolytic cleavage of SREBP 1, which resulted in decreased fatty acid synthase expression. Administration to humans rapidly increases resting metabolic rate. Upon chronic administration (28 d) body weight decreased and metabolic rate increased in a study of just two participants. Although T3- and 3,5-T2-induced effects are inhibited by propranolol, application of deiodinases did not influence the effects and these were more rapid than T3-induced events, suggesting independent mechanisms of T3 and 3,5-T2 actions. T3 stimulates fatty acid synthesis (FAS, fatty acid synthase) and β-oxidation (ACC, acetyl-CoA carboxylase) while 3,5-T2 stimulates only β-oxidation (105). Decrease of FAS is regulated by SREBP-1 at the non-genomic level. 3,5-T2 stimulated mitochondrial oxidative metabolism of fatty acids and reduced SREBP-1. The latter effect was linked to the pro-apoptotic effects of T2, suggesting potential application as an anti-cancer agent. Other effects of 3,5-T2 include activation of protein kinases Akt, p38, PKC-δ, ERK, ATP-activated protein kinase (AMPK) and silent mating-type information regulation 2 homolog (sirtuin, SIRT)1, a nuclear deacetylase that enhances mitochondrial activity.

### Thyronamines

Thyronamines are decarboxylated and deiodinated metabolites of thyroid hormones with 3-iodothyronamine (3-T1AM) and thyronamine (T0AM) as the most important representatives in humans. Blood levels are 0.6-2.3 ng/dl but intracellular levels can be 20 times higher than T3 and twice as high as T4 (106). 3-T1AM is produced by ornithine carboxylase and is very stable. It is observed up to 6 days after oral application of L-T4. 3-T1AM accumulates in skeletal muscle, myocardium>liver>adipose tissue. 3-T1AM and T0AM differ from T3 and T4 regarding their effects and their binding to serum proteins in the way that the highly lipophilic molecules are transported by apolipoprotein B100 instead of the common TH distributor proteins. As a general rule, the action of 3’3,5-T3 is roughly equal to 3,5-T2, while T1AM acts as an antagonist. T3 has a stronger action on body temperature and insulin secretion than 3,5-T2, while the opposite applies to insulin sensitivity, heart rate and contractility (107). Upon activation of 3-T1AM, metabolic rate decreased and metabolism was shifted from carbohydrate to lipid with elevated H_2_O_2_ production. Sirtuin increased expression of SIRT4 and SIRT6-dependent genes. 3-T1AM activates trace amine-associated receptor 1 (TAAR 1), adrenergic receptors, and serotonin 1β receptors and reduces metabolic rate and increases lipid utilization, resembling the pattern of sleep or hibernation. 3-T1AM has also neuromodulatory effects, which are, according to animal experiments, partly mediated by inhibition of dopamine and norepinephrine reuptake and transport into synaptic vesicles. 3-T1AM and T0AM cause similar effects (e.g. hypothermia, negative chronotropy, reduction of respiratory coefficient). More details are given by Köhrle (85).

### Thyroacetic Acids (Tetraiodoacetic and Triiodoacetic Acid)

Thyroacetic acids circulate at relatively high levels in blood and are formed by desamination of iodothyronines. Tetraiodoacetic acid (Tetrac) is a physiological metabolite of T4, a ligand of αvβ3 integrin, and a precursor of triiodoacetic acid (Triac). Levels are increased upon fasting as with T3 and appear to serve the same purpose, i.e. the shunting away of T4 from T3 (108). Half-lives of Triac at 6h and Tetrac at 3-4d are related to the half-lives of T4 (7d) and T3 (1d), respectively. Deiodinases D1 and D2 can convert Tetrac to Triac, and D1 and D3 can deiodinate Triac with higher affinity than T3. The hydrophilic molecules have little binding to the distributor proteins, and are transported exclusively by TTR. Cellular uptake of Triac takes place by tissue-specific transporters different from MCT8, MCT10 and OATP. Triac is excreted in the urine after conjugation to glucuronic acid. Tetrac and Triac are much better substrates for glucuronidation than T4 and T3 and are, as sulfates and glucuronates, better substrates for deiodination in liver and kidney than T3 and T4 (109).

Tetrac blocks T4 at the binding site of the αvβ3 integrin and acts *via* MAPK/ERK signaling in competition with T4. The binding disrupts cross-talk with adjacent growth factor receptors like VEGF, platelet-derived growth factor (PDGF), basic fibroblast growth factor (bFGF), and EGF (110). Tetrac appears to be able to disrupt this cross-talk and through this mechanism block migration, angiogenesis and tissue invasion of cancer cells (111). Tetrac can also lower DNA repair, drug resistance and radioresistance by blocking P-glycoprotein (P-gp) and multidrug resistance protein (MRP). By its action on the Na^+^/H^+^ antiporter, the pH shifts away from the optimum of the P-gp pump and the intracellular pH becomes more acidic. In cancer cells with inherent radioresistance due to changed conformation of the receptor, Tetrac can reduce the effect (96). In addition to prevention of angioneogenesis in cancer, Tetrac can reduce fibrosis in hepatic fibroblasts (35). The increased Tetrac levels in patients with Graves’ disease have to be discussed in the light of the greater risk of these patients for cancer (112) and the promoting effect of hyperthyroidism on cancer progression (36, 113). It may be speculated that conversion of T4 to Tetrac in cancer patients is lower than the 20% reported in normal individuals (114). In this way the effects of the other TH may outbalance the anti-tumor effects of Tetrac.

Similar to rT3, Triac levels are increased in fasting and in NTIS. However, in contrast to rT3, Triac acts as a thyromimetic, affecting the liver, adipose tissue, bone, and brain but not the heart. Triac binds to TRα1 with similar binding affinity as T3 and to TRβ with six-fold higher affinity but has limited potency due to its short half-life (115). Triac causes pituitary suppression of TSH, and effects on bone, kidney, liver and body weight similar to T3. Triac has suppressive function on TSH and decreases leptin secretion by adipocytes in this condition. This is surprising because, when blood T3 levels are low, increased TSH is expected. Triac is special because the metabolite acts as a thyromimetic in hypothyroid conditions and increases metabolism. In the euthyroid condition, metabolic rate is reduced. The applied doses were 4-30 times lower than L-T4 and 15-100 times lower than L-T3 but much higher than needed for TSH suppression.

## Medical Uses of Iodothyronines, Thyroamines, and Iodoacetates

Medical uses may consist in functioning as biomarkers or as candidates for treatment.

### Use of L-T4 and L-T3

Thyroxine (T4) is one of the top 10 medically-prescribed drugs worldwide, with the most common indication being hypothyroidism. At first glance, supplementation of TH appears less challenging than application of insulin in diabetes mellitus, where food intake, physical activity and stress change hormone requirement (116). Levels of T3 have a diurnal rhythm with peak round 4 am and a nadir between 3-5 pm but actual differences in T3 are low (117). T4 levels are constant mainly due to the long half-life of T4 of about 7 days, which eliminates the need to adapt for circadian changes. On the other hand, identification of the ideal personalized dose may pose problems. Requirements of L-T4 appear to be influenced by residual thyroid function because the small amounts of co-secreted T3 upon normal T4 secretion have regulatory function on the HPT axis (118).

Doses for L-T4 supplementation can be calculated based on body weight or body mass index (BMI) with and without inclusion of additional factors (e.g. patient sex). Generally, both a TSH-based estimate and a body weight-based estimate yield similar initial estimates of dose requirement (119). Type of formulation, co-medication (iron, calcium carbonate, -citrate, -acetate, vitamin C, phenytoin, rifampin) and nutrients (soja products, milk, coffee, grape fruit, papaya), co-existing conditions (pregnancy, renal failure, hepatic disease), deiodinase expression and peripheral conversion, dosing (lean/obese), drug distribution (circulation, plasma proteins), drug absorption (food intake, timing gastrointestinal tract motility), patient compliance, co-morbidities (malabsorption, surgery), and disease stage (stable, progressing, residual thyroid tissue) cause variable TH levels upon administration of the same amount of L-T4 (120). Conversely, chronic conditions like diabetes mellitus, cardiac disease, hepatic disease, osteoporosis do not have a pronounced influence on L-T4 requirement. Some general rules, the reduced requirement of aged individuals and postmenopausal women should also be taken into account. A list of medical conditions, food ingredients, and drugs that interfere with L-T4 supplementation is available elsewhere (121). If patients complain of hypothyroid symptoms despite L-T4 therapy, a panel of other diseases, which could cause symptoms similar to TH deficiency, has to be excluded. These conditions include: diabetes mellitus, adrenal insufficiency, hypopituitarism, celiac disease, pernicious anemia, anemia, multiple myeloma, chronic kidney damage, chronic liver disease, and congestive heart failure (117). Similarly, B12 deficiency, folate, vitamin D, and iron deficiency, obesity, hypercalcemia, electrolyte imbalance, treatment with β-blockers, statins and opiates, stress, lack of sleep, alcohol excess, sleep apnea, chronic fatigue syndrome, co-poisoning, depression, polymyalgia rheumatica, and fibromyalgia can mimic inadequate L-T4 supplementation. Reasons for insufficient treatment like lack of compliance or change of L-T4 formulation should also be considered. The different existing formulations were regarded as bioequivalent and products, in theory, should be interchangeable (122). Bioequivalence of two products means that 90% confidence interval of the ratio of the log-transformed exposure measure, area under the curve (AUC) and maximal plasma concentration (Cmax), falls within 80-125%. The method is not undisputed because only T4 levels are used for the evaluation and conventional immunoassays may overestimate TH levels. Furthermore, the method cannot distinguish dose differences of up to 33% (e.g. 400 *vs* 600 µg) and doses that differ by 12.5% (400 *vs* 450 µg). There is uncertainty if products are really bioequivalent and, therefore, change between products is not recommended because changes of 12.5 µg may have dramatic effects on a drug like L-T4, which has a narrow therapeutic window. In gastrointestinal diseases such as celiac disease, gastritis, or lactose intolerance, that decrease absorbance, liquid formulations may work better than tablets. However, even when euthyroidism according to standard readout parameters for TSH and fT4 is achieved, ~15% of patients experience some level of psychological impairment. These patients express the wish for alternative treatment, often the combination of L-T4 with L-T3 or the prescription of desiccated porcine thyroids. The D-enantiomer of L-T4 dextrothyroxine (D-T4) has been tested in clinical trials for its antihyperlipidemic effects. Although D-T4 lowered serum cholesterol in the Coronary Drug Project (1966 and 1975), groups were discontinued early because of increased mortality, probably due to tachycardia (123).

The question whether combined L-T3 + L-T4 supplementation is superior to L-T4 monotherapy has been under debate for many years and numerous reviews have been dedicated to this question (e.g (4, 124, 125).). Patients under replacement therapy have lower T3 and higher T4 levels compared to control individuals. When L-T4 was dosed to reach T3 levels of the controls, TSH was lowered or suppressed (126). Only the combination of L-T3 + L-T4 normalized TSH, serum and tissue T4 and T3 concentrations. In another study including patients with elective total thyroidectomy, L-T4 monotherapy was able to bring serum T3 back to the same presurgical levels without suppressing TSH but with elevated fT4 levels. Also better quality of life under combination therapy compared to L-T4 monotherapy has been reported (127). Improved effects by L-T3 + L-T4 combination may be explained by placebo effects, differential signaling in neurons and positive effects by action on serotonin and catecholamines (120). A systematic review of ten randomized controlled trials comparing combination therapy *vs* monotherapy found no statistically significant differences in biochemical markers, mood states, and adverse effects. Commercially available products like Armour Thyroid, Nature-Throid, Bio-Throid, WP-Thyroid, Westhiod, and NP-Throid consist of desiccated porcine thyroid extracts. Although containing both TH may be advantageous, one major criticism is the species-specific relation of T4:T3, of 4:1 in pigs and 14:1 in humans. Combination therapy may only be adequate in patients bearing the Thr92Ala D2 (rs225014) polymorphism. This genetic polymorphism may affect half-life of the protein and could hypothetically disrupt TH signaling in D2-expressing tissues. Nevertheless, human studies, so far, did not find lower TSH levels, changes in fT4 and fT3, metabolic syndrome etc. in carriers of this polymorphism (119). Patients with MCT10 (rs17606253) polymorphism, on the other hand, preferred the combination therapy to L-T4 monotherapy (128). This led to the recommendation to restrict the combination therapy to populations with such polymorphisms. T3 supplementation is adequate for patients with TR mutations, where TRβ mutation hyperthyroid symptoms are frequent upon L-T4 therapy. They manifest as tachycardia because heart effects are dominated by TRα signaling and when the L-T4 dose, which is adjusted to the dysfunctional TRβ is too high (129). In patients with TRα mutations, low T4/T3 ratio is typical and symptoms of hypothyroidism with variable manifestations are seen. The consensus report on evidence-based use of L-T3/L-T4 combinations in treating hypothyroidism reported that there is dissatisfaction with the existing standard of care and that “a new well-designed adequately powered clinical trial of combination therapy” is needed because not all studies met the current standard requirements (130).

Treatment of heart diseases may be another indication for use of TH. This may appear surprising as arrhythmia and mortality are linked to overdoses of L-T4. However, in heart disease, tissue levels of deiodinases are altered and less T3 is being produced. In hypothyroidism MCT10 expression in the heart is increased and deiodinase expression thus changed to produce more T3. The opposite is observed in hyperthyroidism showing that the heart is capable to adapt to abnormal TH levels. A combination of L-T4 + L-T3 is suggested because supplementation with either of the TH alone did not restore T3 in rats (131). L-T3 alone has positive effects in myocardial infarction and protects hypothyroid rats against arrhythmia. L-T3 and L-T4 showed beneficial effects in patients with heart failure (132). Small clinical trials reported promising effects of L-T3 administration in patients with acute myocardial infarction, cardiac surgery and transplantation.

TH interact with catecholamines (epinephrine and norepinephrine) in that infusion of T3 before trauma in pigs increased epinephrine levels and decreased T3 and T4 levels (133). The resultant hypothyroid state decreased responsiveness to α- and β-adrenergic agonists. Similar effects occur also during brain death and provide the theoretical basis for the use of TH in preparation of organ transplantation. It is assumed that loss of TH, cortisol, antidiuretic hormone (ADH) and insulin leads to reduction in perfusion, inhibition of mitochondrial function and organ failure. Therefore, the United Network for Organ Sharing (UNOS) Critical Pathway for the Organ Donor recommends hormone replacement with TH, vasopressin, methylprednisolone and insulin; both L-T3 and L-T4 may be used. According to a large retrospective analysis L-T3 or L-T4 therapy produced more transplantable hearts, lungs, kidneys, pancreases, and intestines, while the number of transplantable livers was not increased (134). Positive effects include increased chronotropy and upregulation of ATPase-rich α heavy chain isoenzyme that results in increased inotropy. In combination with the increased smooth muscle relaxation and vasodilation, diastolic pressure is lowered and resistance to left ventricular ejection reduced (135). In general, disparate results were reported for heart transplantation. The studies differed in the use of TH (either alone or in combination with other hormonal therapies) in methodology and biological readouts. For instance, donor administration of L-T4 improved survival of the graft recipient. The recipients who received L-T4 prior to the transplantation had a survival advantage over those who did not and results were better when L-T4 was applied to the donor before the declaration of brain death (136). Early L-T4 therapy was associated with an increase in solid organ procurement rate with odds ratio of 1.9 (137).

Application of L-T3 alone is commonly restricted to specific situations such as preparation of patients with advanced thyroid cancer for nuclear imaging, for primary ablation or whole body scan, if recombinant TSH is not available. Further, clinical data indicate that application of L-T3 to patients in the terminal phase of lung cancer, pancreatic cancer, mesothelioma, soft tissue cancer, and glioblastoma can prolong survival (138). The interventional lowering of fT4 by application of L-T3 alone or in combination with methimazole is better tolerated than withdrawal of TH by methimazole alone. Twenty percent of the patients exceeded the 20% expected 1-year survival upon L-T3 treatment (139). The reported prolongation of survival may be caused by reduction of the promoting effects of T4 on tumor proliferation, coagulation, and angiogenesis. Additional indications appear possible as L-T3 in septic rats prevented the consumption of coagulation inhibitors like ATIII (140).

Efficacy of L-T3 in critically ill COVID-19 patients is under investigation. A phase II clinical trial (ClinicalTrials.gov Identifier: NCT04348513) including patients diagnosed with pulmonary infection due to COVID-19 in intensive care requiring mechanical respiratory support was started. The trial will study the effect of intravenous high dose L-T3 for enhancing recovery of critically ill COVID-19 patients. In this trial, T3 treatment is started with a bolus injection, followed by maintenance dose of 0.113 g/kg/h for 38h and 0.057 g/kg/h for up to 30d (141). Successful weaning from ventilation was defined as primary outcome. The treatment was initiated under the assumption that thyroid dysregulation in severely ill COVID-19 patients shows the pattern of NTIS. Although a correlation between low T3 and diseases severity has been established, replacement with TH in NTIS in general is not suggested (142). Application of L-T3, instead of L-T4, was chosen based on the following findings; i) T3, in contrast to T4, does not induce hypercoagulation (71), ii) T3 stimulates secretion of the anti-inflammatory IL-10 and strengthens specific immune response by action on DCs (79), and iii) T3 prevents action of T4 on integrin αvβ3. T4 may be facilitating virus uptake because it increases expression of the genes for the specific integrin monomers αv and β3 and increases internalization of the integrin (143). In other words, fT4, in contrast to T3, may increase the number of binding sites for the virus on the target cell surface. Finally, endothelial dysfunction, induced by increased TSH level, may improve because high T3/T4 levels will reduce TSH secretion of the anterior pituitary gland (144). In the light of later studies, which reported heterogenous pattern of thyroid dysfunction with decreased TSH levels in combination with decreased T3 or increased T4 levels (**Table 1**), systemic administration of L-T3 appears debatable. The differentiation between decreased TSH values as indication for NTIS in prolonged disease or as thyrotoxicosis is very important because 32% of thyrotoxic COVID-19 patients developed atrial fibrillation (59). Atrial fibrillation is not typical for viral SAT but one of the most common arrhythmias caused by SARS-CoV-2-induced myocardial injury (58). It was hypothesized that the SARS-CoV-2 virus showed specific cardiotoxicity but a more recent study suggests that patients with pre-existing heart disease may be more likely to contract the disease (145). In hyperthyroidism, T3 more than T4, may cause atrial fibrillation (146). It is, therefore, possible that a combination of L-T3 administration and COVID-19 induced myocardial damage leads to an increase in adverse cardiac effects. The determination of physiological parameters and troponin I levels performed in the NCT04348513 trial are suitable to identify such effects. Another phase II trial will study effects of locally administered T3 to reduce pulmonary edema in COVID-19 pneumonia (ClinicalTrials.gov Identifier: NCT04725110), based on beneficial effects of intratracheally administered modified Triostat^®^ (liothyronine) to rats on alveolar fluid clearance (147). Primary outcome is the change of the extravascular lung water index. Kidney function (glomerular filtration rate and creatinine levels) but not cardiac function will be monitored. Although local action on the Na/K ATPase to stimulate alveolar clearance is the primary mode of action (148), the lipophilic molecule can also cross the alveolar barriers and reach the systemic circulation, resulting in increases of circulating T3 levels.

L-T3 is more difficult to dose correctly than L-T4 because absorption is fast and half-life is short. Absorption leads to 40% increase in T3 blood levels, which is markedly higher than the normal daily fluctuation of 5-10%. The high T3 levels represent a risk factor for cardiovascular events and hip fractures (149). Slow release formulations, such as metal coordinated poly-zinc-liothyonine, may be a solution because stable levels of circulating T3 were obtained in rats. T3 from the poly-zinc complex is slowly absorbed. Over a time span of 8 days levels gradually increased but were still in the reference range. Alternatively, T3 sulfate may be applied, which has to be activated in the liver. Oral administration of T3 as sulphate may also be an option because endogenous desulphatases can slowly produce T3 (150).

Adequate supplementation with L-T4 is monitored by levels of TSH, fT4, fT3 and fT4/fT3 ratio. It is agreed that fT3 is a much better biochemical marker for euthyroidism than TSH but determination of fT3 levels is often inaccurate (118). A recent meta-analysis reported that fT4 levels were better correlated to clinical symptoms than TSH levels (151). There is no general recommendation by the European Thyroid Association to determine rT3 levels, except in infantile hepatic hemangiomatosis (IHH). This condition is also termed consumptive hypothyroidism and has to be differentiated from congenital hypothyroidism because treatments differ (152). Consumptive hypothyroidism in adults due to hemangioma or hemangioendothelioma has only been reported in a few case studies (99). Although both diseases need supplementation with TH, patients with IHH receive 22−70 μg/kg/day L-T4 or L-T3 compared to 5−10 μg/kg/day L-T4 or L-T3 in congenital hypothyroidism. For combinations of L-T4 and L-T3, lower doses may be required (153). The reason for the thigh doses in IHH is the high expression of D3 in the hemangioma tissue. It has been postulated that IHH may originate from placental angioblasts and arise from embolization of placental endothelial cells. The liver carries the highest risk for hemangioma development as it is the first organ to be perfused with the incoming blood from the placenta (154).

### Use of 3,5 T2

3,5 T2 is an interesting candidate as hypolipidemic drug and for cancer therapy. 3,5-T2 has a suitable action profile for targeting steatosis because it acts independently of T3. Anti-steatotic effects of T3 and 3,5-T2 are caused *via* different mechanisms. 3,5-T2 decreases lipogenesis and increases β-oxidation, while T3 only increases β-oxidation. TSH suppression and cardiac effects were observed. In summary, studies differed in dosage of 3,5 T2 (10-100 µg/kg), treatment regime (single versus chronic) and application route (sc or ip). High fat-induced obesity with co-administration or treatment with 3,5-T2 did not show anti-steatotic effects in all studies (155). Accumulation of 3,5-T2 in the liver was seen only upon application of this molecule, while no accumulation of 3,5-T2 was observed when T3 was applied. The effect was specific for hepatocytes and did not occur in cardiomyocytes. 3,5-T2 increased glucose consumption but did not increase inotropy and chronotropy. In skeletal muscle ATP kinase activation and increased GLUT4 expression induced a switch from fast-twitch (white fibres) to slow-twitch (red fibres) typical for glycolytic phenotype. In animal studies no clear evidence was obtained that the anti-steatotic effects occur at concentrations that do not suppress the HPT-axis or cause adverse effects on the heart. Similarly, in a 4-week trial with the 3,5-T2-mimetic analogue TRC-150094 no increase in insulin sensitivity, decrease of fatty acids in serum or in intrahepatic triglycerides was observed (156). Based on this data the use of 3,5-T2 for treatment of metabolic syndrome and for reduction of body weight is discouraged. This applies also for use outside of the medical setting, where individuals who wish to lose weight or boost their energy use 3,5-T2 as a dietary supplement. These over-the-counter products contain variable (50-300 µg/pill) and often not stated amounts of 3,5-T2 (157).

Other molecules having the same profile as 3,5-T2 have been designed to act preferentially on TRβ receptors, expressed in liver and brain, and to avoid cardiac adverse effects induced by binding to TRα. Some of these molecules were designed based on the finding that the binding pocket of TRβ is more flexible than that of TRα (32). GC-1 (sobetirome) and KB-2115 (eprotirome) had a 10- and 20-fold higher TRβ/TRα ratio than T3, respectively. KB07811 (VK2809) is a prodrug converted to the active compound in the liver, and MGL-3196 (resmetirom) has a 30-fold higher TRβ/TRα ratio than T3. Studies for the indication of hypercholesterolemia were successful but stopped because unexpected cartilage defects in canine bones were seen in preclinical testing (158). For application in non-alcoholic fatty liver disease, however, MGL-3196 has been tested in clinical trials (159), and results are encouraging. Similarly, also VK2809 reduced liver fat content in clinical trials. Another indication for sobetirome is treatment of X-linked adrenoleukodystrophy (ALD), where the protocol of the planned clinical trial is currently being revised (32). The disease is caused by mutation of very long fatty acid transporters located in the peroxisomal membrane encoded by the *ABCD1* gene.

### Use of Thyroamines

3-T1AM has several biological effects in animals, where it increases plasma glucose, induces carbohydrate oxidation, gluconeogenesis, ketogenesis and decreases body weight in obese mice (160). In addition to the endocrine effects, 3-T1AM favors learning in animals by action on TAAR1 (161). The molecule, however, has a very short half-life because sulfatation and glucuronidation are so fast that very frequent doses are needed to obtain the desired effect. By its negatively inotropic and chronotropic action it can prevent myocardial lesions caused by ischemia. It binds to transient receptor potential cation channel subfamily melastatin member (TRMP) 8 that blunts function of the Ca-channel Transient Receptor Potential Vanilloid (TRPV4) expressed by tumor cells and activated by heat, capsaicin, binding of growth factors and cytokine secretion. Hypothermic effects, modulation of glucose and insulin secretion, and cardiac effects suggest potential application in myocardial infarction and brain ischemia (85). Effects of 3-T1AM on feeding behaviour, learning, anti-amnestic response, protection against β-amyloid toxicity, and anticonvulsant effects, observed in rodents, present interesting applications but have not been studied in humans so far (32).

### Use of Thyroacetic Acids

The two thyroacetic acids differ markedly in their action, Triac as a thyromimetic and Tetrac as a TH antagonist. The advantage of Triac from the pharmaceutical perspective is the fact that it accesses the brain by bypassing MCT8 (85). On the other hand, Triac is very rapidly conjugated to glucuronic acid (~1500 and ~200 times faster than T3 and T4, respectively) and needs frequent application.

The main off-label application of Triac is treatment of patients with TRβ mutations (115). AHDS may be another indication for Triac to prevent the local hypothyroidism in the brain due to MCT8 mutation. Following promising results in animal models, a clinical trial in children harboring mutations in MCT8 has been initiated. Triac can be purchased over the counter in several European countries as a dietary supplement (trade name: Tiratricol) to reduce weight. Anti-angiogenetic effects may occur but were never reported by users. The FDA issued an official warning not to use Triac-containing supplements because Triac markedly reduced bone density and may induce thyrotoxic hypokalemic periodic paralysis (162).

Tetrac, when taken up into cells acts as a weak thyromimetic. In order to cause effects mainly be αvβ3 integrin signaling to improve anti-tumor action, increase of the extracellular action of Tetrac is important. For pharmaceutical application in cancer, Tetrac was bound to 200 nm poly(lactic-co-glycolic acid) (PLGA) nanoparticles (Nanotetrac) and the binding resulted in increased antiangiogenic efficacy *in vitro* (71). After the uptake, nanoparticles were located in the cytoplasm and Tetrac in the nucleus of cancer cells. Action was caused by regulation of cytokines in combination with maturation of endothelial cells, stabilization of vessels, and inhibition of neo-angiogenesis. *In vivo*, Tetrac antagonized T3 and T4 effects regarding differentiation of mesenchymal stem cells (MSCs) into cancer-associated fibroblasts, angiogenesis and recruitment of MSCs to tumor cells. In animal models Nanotetrac was 10-times more efficient in inhibiting tumor growth than the original molecule (163). Tetrac was granted Orphan Drug status by the US FDA to be used in thyroid cancer treatment (164).

## Conclusions

The action of TH is more complex than that of other endocrine amine hormones. Endocrine action of catecholamines for example has many targets to increase blood pressure, heart rate, glucogenolysis, glucagon secretion, and decrease insulin secretion and lipolysis but acts only by the two hormones epinephrine and norepinephrine (165). In contrast, five metabolites have agonistic and antagonistic effects to the classical TH T4 and T3.

**Figure 5:**
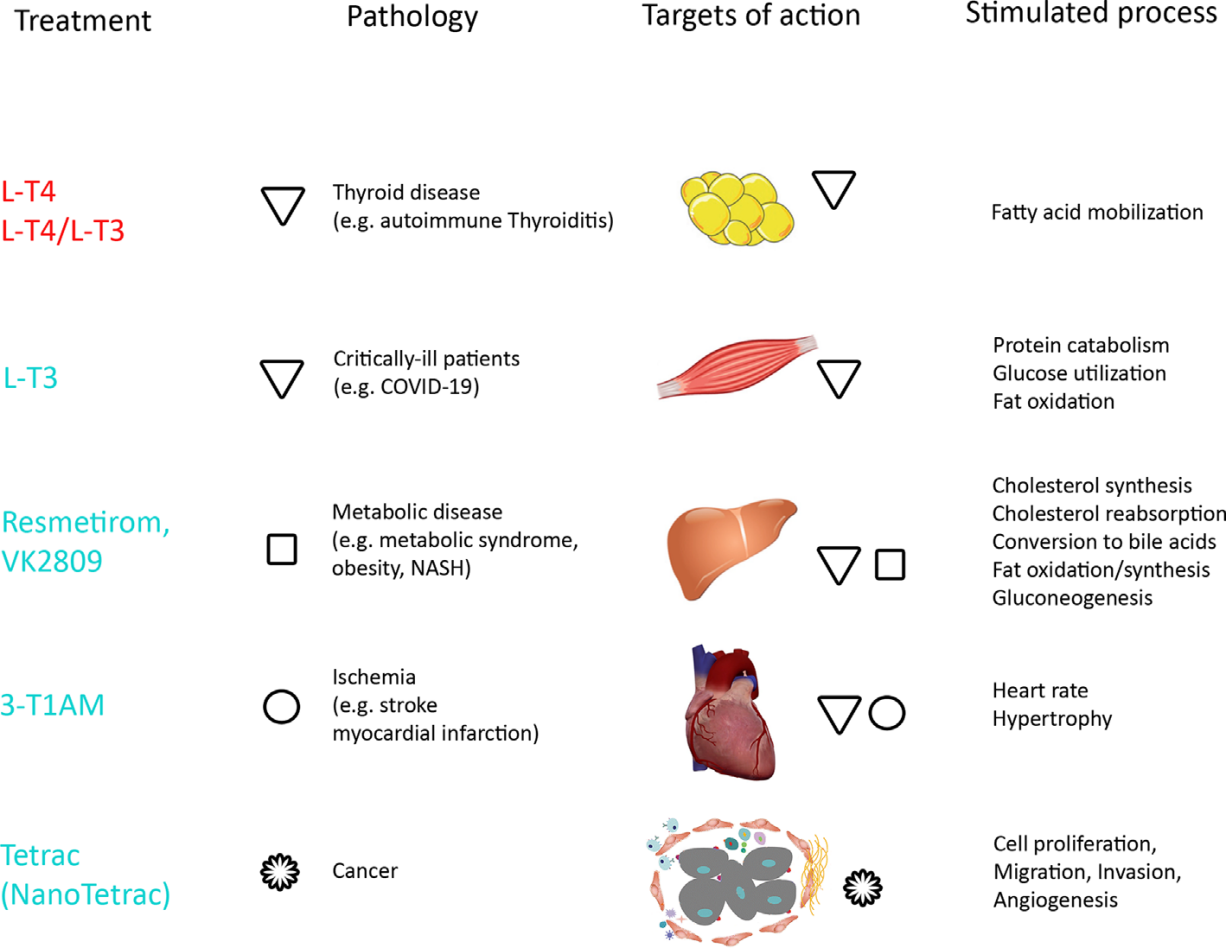
Overview of treatments, pathologies and targets of action with stimulated processes by TH. Tissues relevant for the review are shown and other target tissues like bone, brain, kidney, gastrointestinal tract not represented. Established treatments are written in red, compounds/applications in clinical trials in turquoise. Pathologies and affected tissues are marked by symbols for the compounds (triangles for T3 and T4, squares for Resmetirom and VK2809, circles for 3-T1AM, and rosettes for Tetrac).

A comparison of biologically active TH metabolites shows that i) the more potent acting T3 and Triac appear to have shorter half-lives than less potent antagonists 3T1AM and Tetrac. Ii) rT3 and 3,5-T2 may serve as indicators for metabolic dysregulation and disease, and iii) Nanotetrac may be a promising candidate for treatment of cancer and MGL-3196 for steatohepatitis, obesity, and metabolic syndrome (**Figure 5**). Use of L-T3 or combinations of L-T3 and L-T4 appear not to act better than L-T4 in common indications (e.g. hypothyroidism or thyroidectomy).

The observed alterations of TH levels in COVID-19 may result from a combination of thyrotoxic effects and NTIS. Outcome of local and systemic administration of L-T3 in critically ill patients potentially may provide further insight into the still unclear relationship between SARS-CoV-2 and thyroid metabolism.

## Author Contributions

All authors listed have made a substantial, direct and intellectual contribution to the work, and approved it for publication.

## Conflict of Interest

The authors declare that the research was conducted in the absence of any commercial or financial relationships that could be construed as a potential conflict of interest.
