# Supplementary figures and images for: Physiological Role and Use of Thyroid Hormone Metabolites - Potential Utility in COVID-19 Patients
Source: Front Endocrinol (Lausanne). 2021 Apr 26;12:587518. doi: 10.3389/fendo.2021.587518 (PMC8109250; doi:10.3389/fendo.2021.587518)

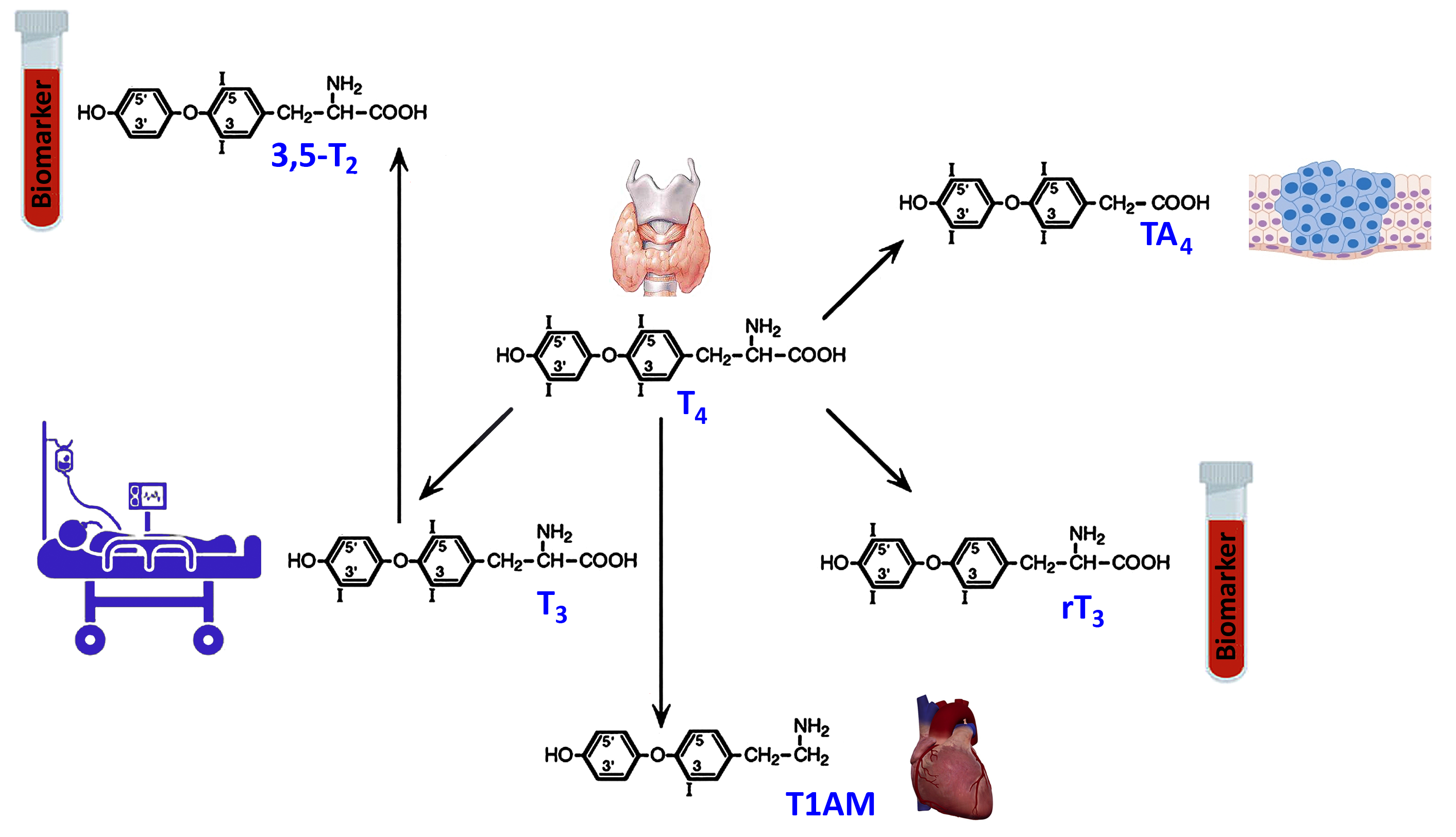

Supplement: Supplementary file 1 [file Image_1.tif]
